# A new iphiculid crab (Crustacea, Brachyura, Leucosioidea) from the Middle Miocene of Austria, with notes on palaeobiogeography of *Iphiculus*

**DOI:** 10.11646/zootaxa.4179.2.6

**Published:** 2016-10-31

**Authors:** Matúš Hyžný, Martin Gross

**Affiliations:** 1 Comenius University, Faculty of Natural Sciences, Department of Geology and Palaeontology, Mlynská dolina, Ilkovičova 6, SVK-842 15 Bratislava, Slovakia; 2 Geological-paleontological Department, Natural History Museum, Vienna, Burgring 7, A-1010 Vienna, Austria; 3 Department for Geology & Palaeontology, Universalmuseum Joanneum, Weinzöttlstrasse 16, 8045 Graz, Austria

**Keywords:** Decapoda, Iphiculidae, new taxon, Badenian, Styrian Basin

## Abstract

A new fossil species of the iphiculid genus *Iphiculus* Adams & White, 1849, (Crustacea, Brachyura) is described on the basis of three specimens from the Middle Miocene Florian Beds of Styria, Austria. *Iphiculus eliasi* **sp. nov.** constitutes the first European record of the genus. This occurrence represents the oldest record of *Iphiculus*, having implications for the palaeobiogeographic history of the family Iphiculidae. It is suggested that *Iphiculus* may have originated in the Western Tethys and migrated subsequently into the Indo-West Pacific. Alternatively, its current geographic restriction to the Indo-West Pacific can be a remnant of an ancient broader geographic distribution.

## Introduction

The fossil record of leucosioid crabs is relatively rich, and more than 100 species have been described to date (Schweitzer *et al.* 2010). Following Ng *et al*. (2008) and Artal & Hyžný (2016), three distinct families are currently recognized: Leucosiidae Samouelle, 1819, Iphiculidae Alcock, 1896, and Folguerolesiidae Artal & Hyžný, 2016, the latter with only fossil representatives. Most of known fossil taxa belong to Leucosiidae and only nine iphiculid crabs have been reported from the fossil record, all of them from the Indo-West Pacific (Table 1). The monotypic family Folguerolesiidae is exclusively known from the Eocene of Spain (Artal & Hyžný 2016). A new species of *Iphiculus* Adams & White, 1849 is herein described from the Miocene of Austria. This occurrence constitutes the first fossil record of the genus from Europe. It represents also the oldest record of the genus and as such it has implications for the palaeobiogeography of the family.

## Material and methods

**Figure 1:**
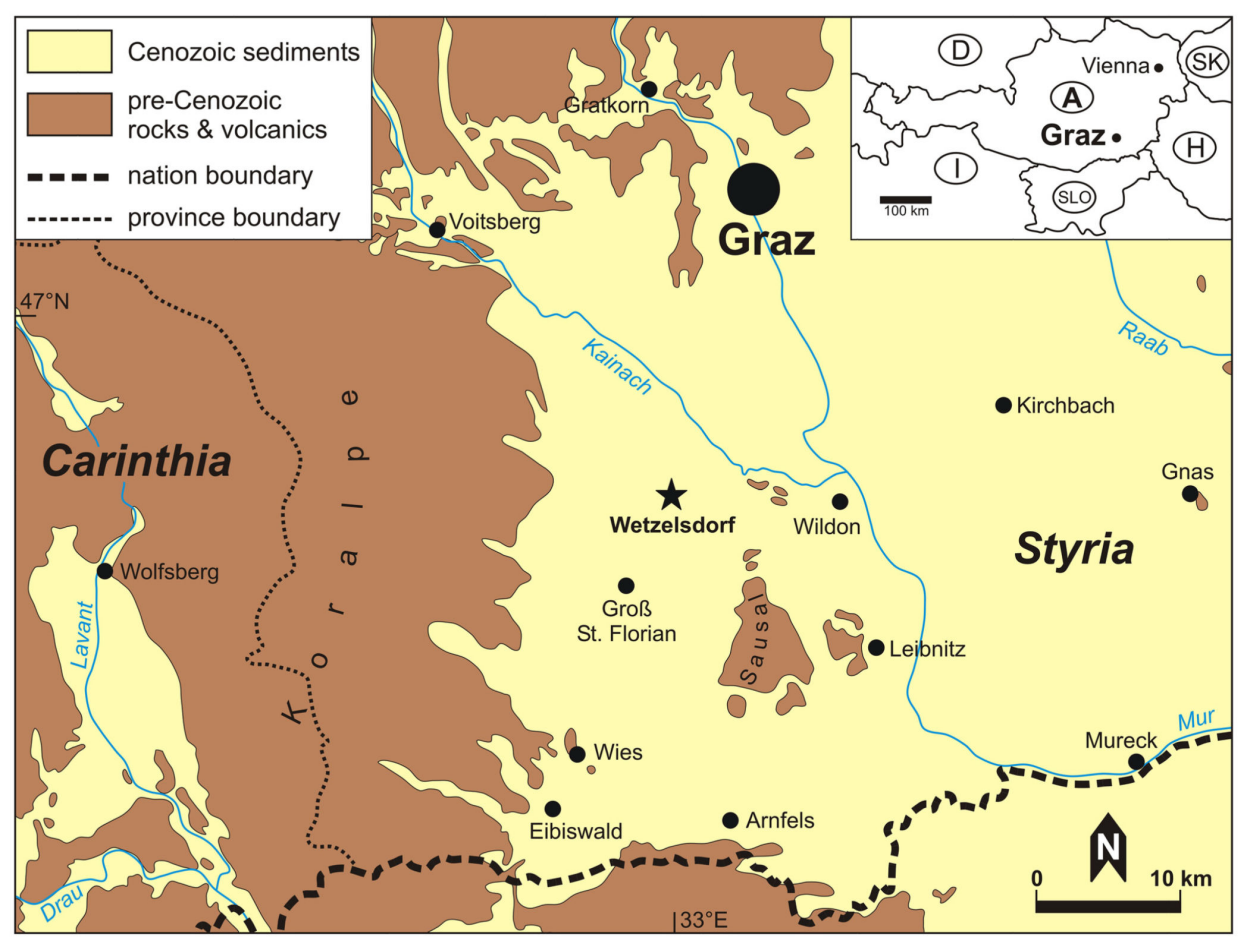
Study area with the type locality of *Iphiculus eliasi* **sp. nov.** indicated (asterisk).

Fossil material was recovered from the region of Wetzelsdorf, Styria, Austria (Fig. 1). Several sites furnished quite a diverse brachyuran fauna (13 species; Hyžný & Gross in press). One of these sites yielded several carapaces that form the basis of this study. At the locality the Middle Miocene (Lower Badenian) Florian Beds (*Florianer Schichten*) are exposed (Holler 1900; Kopetzky 1957; Kollmann 1965; Friebe 1990; Hohenegger *et al.* 2009, 2014).

Specimens were photographed dry and uncoated or coated with ammonium chloride sublimate prior the photography (see figure captions for details). A Leica M205C with the camera DFC290 was used for detailed photography of the carapace surfaces. Extant material of *Iphiculus convexus* Ihle, 1918 (photographs of a male specimen from Vanuatu, ZRC 2009.0462, max. width = 29.0 mm, max. length = 22.0 mm) was examined for comparative reasons.

The following abbreviations are used: UMJGP, Universalmuseum Joanneum, Department for Geology & Palaeontology, Graz, Austria; ZRC, Zoological Reference Collection, Lee Kong Chian Natural History Museum (formerly Raffles Museum of Biodiversity Research), National University of Singapore, Singapore.

**Systematics**

**Infraorder Brachyura Latreille, 1802**

**Section Eubrachyura de Saint Laurent, 1980**

**Subsection Heterotremata Guinot, 1977**

**Superfamily Leucosioidea Samouelle, 1819**

**Family Iphiculidae Alcock, 1896**

***Iphiculus*Adams & White, 1849**

Type species. *Iphiculus spongiosus* Adams & White, 1849, by monotypy.

**Remarks.** A handful of species are currently included in this genus. Its identification in the fossil record (Morris & Collins 1991; Collins *et al.* 2003) is largely based on the carapace outline, configuration of spines on the lateral margins and carapace surface ornamentation. In this respect it should be noted that some species of *Typilobus* Stoliczka, 1871, a leucosiid genus known exclusively from the fossil record, resemble extant representatives of *Iphiculus*. Artal & Hyžný (2016) presented an appraisal of the fossil leucosiid genus *Typilobus*. They pointed out the heterogeneity of the taxon, as previously emphasized by Vía Boada (1969), Müller (1993), and Feldmann *et al.* (2011) and reassigned *Typilobus boscoi* Vía Boada, 1959 to a new genus and family. More re-assignments of species now classified within the broadly defined *Typilobus* are likely once the genus is reviewed. It is possible that some of them will fall within the range of characters which now define *Iphiculus*.

**Figure 2:**
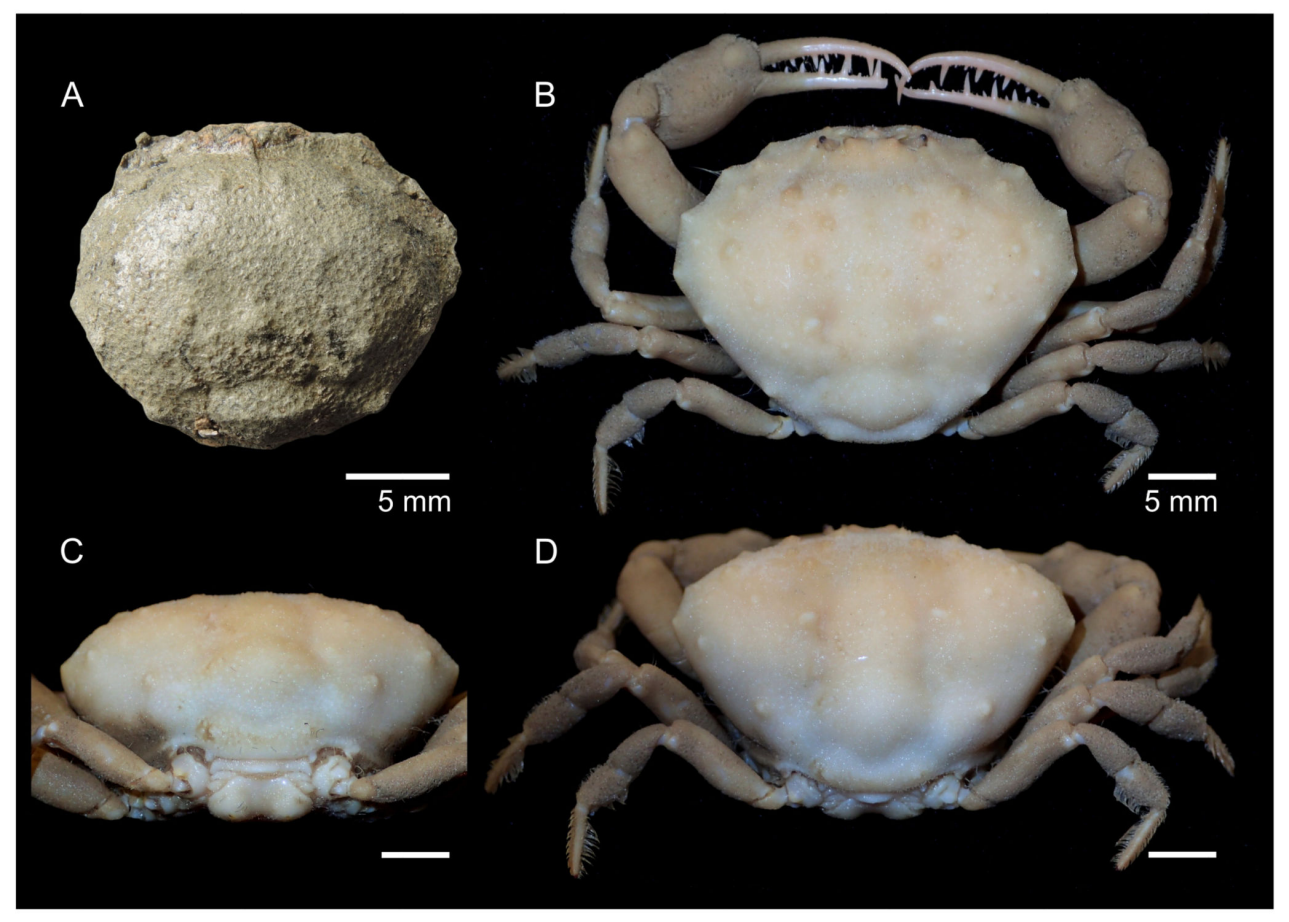
Fossil and extant *Iphiculus*. A, *Iphiculus eliasi* **sp. nov.**, holotype UMJGP 75.612 in dorsal view (dry and uncoated); B–D, *Iphiculus convexus* Ihle, 1918, ZRC 2009.0462 (male specimen from Vanuatu) in dorsal (B), posterior (C), and posterodorsal view (D). Photos in B–D by P.K.L. Ng.

Current assignment of a newly described species to *Iphiculus* is based on striking morphological similarities with extant *I. convexus* (Fig. 2), especially on the general shape of carapace, short anterolateral spines and large rounded tubercles covering evenly the dorsal carapace surface. Since, the new fossil material consists only of isolated carapaces, comparison with sternum, pleon and chelipeds is not possible.

**Figure 3:**
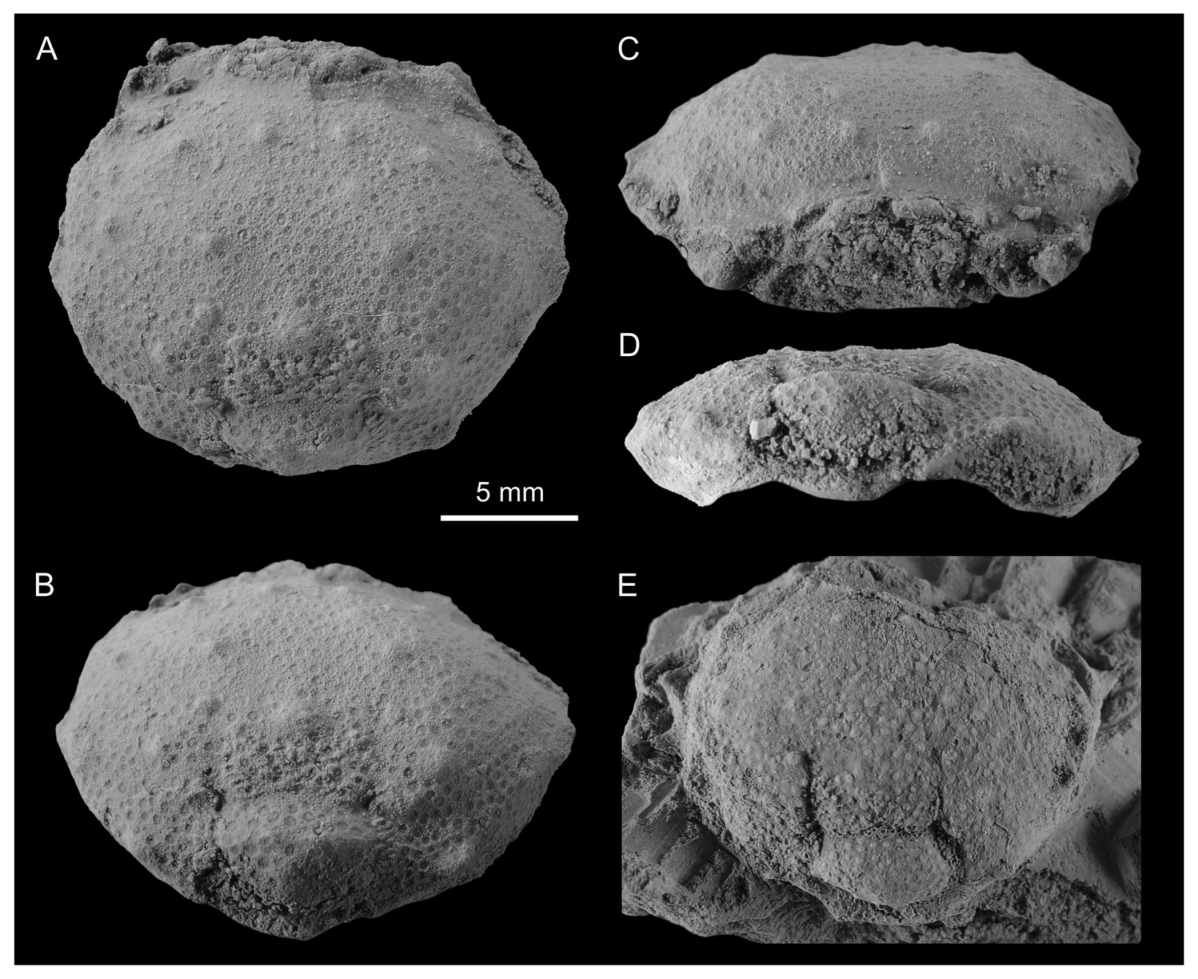
Type material of *Iphiculus eliasi* **sp. nov.** A–D, holotype UMJGP 75.612 in dorsal (A), posterodorsal (B), frontal (C), and posterior view (D); E, paratype UMJGP 75.613 in dorsal view. Specimens were coated with ammonium chloride prior the photography. All specimens to scale.

**Figure 4:**
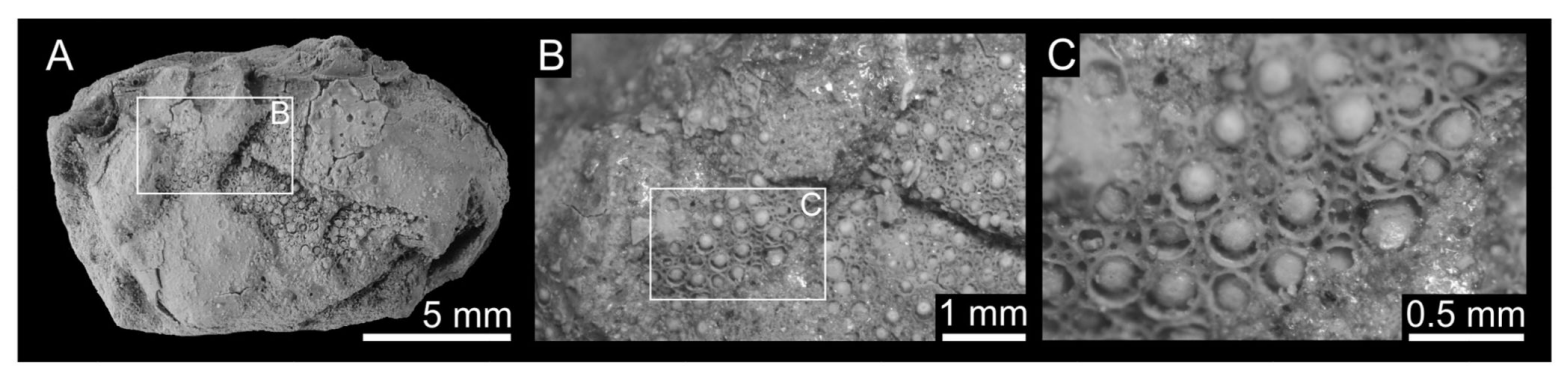
Additional material of *Iphiculus eliasi* **sp. nov.** A–C, UMJGP 21.1339 with partially degraded cuticular surfaces (B–C). Specimen in A was coated with ammonium chloride prior the photography.

***Iphiculus eliasi* sp. nov.**

(Figs 2A, 3A–E, 4A–C)

**Diagnosis.** Carapace transversely subovate in outline, widest at level of posteriormost anterolateral spine; lateral margins bearing 6 short triangular spines; dorsal carapace surface covered evenly with large rounded tubercles: hepatic region with tubercle, gastric region with 3 pairs of tubercles, branchial region with 3 tubercles.

**Etymology.** The species name is dedicated to Eliáš, son of the first author.

**Material examined.** Holotype: near-complete carapace UMJGP Inv.No. 75.612, max. length, 15.7 mm; max. width, 19.0 mm (Figs 3A–D). Paratype: near-complete carapace UMJGP Inv.No. 75.613, max. length, 13.0 mm (preserved portion); max. width, 17.2 mm; Fig. 3E). Additional specimen: carapace fragment UMJGP Inv.No. 211339; Fig. 4A–C).

**Description.** Small carapace; transversely subovate in outline; slightly wider than long, widest at midlength, at level of posteriormost anterolateral spine; dorsal surface moderately convex in both directions. Front not projected, slightly raised, narrow, not well preserved, presumably bilobed. Orbits small, concave, anteriorly directed. Lateral margins bearing 6 short triangular spines, anterolateral margin with 4 spines, posterolateral margin with 2 spines; corners between posterolateral, posterior margins pointed; posterior margin straight, narrow. Dorsal surface of carapace evenly covered with many large, densely packed granules, nearly identical in size (if cuticular surface preserved) or with round concave pustules (if cuticular surface not preserved). Carapace surface covered evenly with large rounded tubercles: hepatic region with 1 tubercle, gastric region with 3 pairs of tubercles, branchial region with 3 tubercles. Carapace grooves absent in anterior carapace portion, well developed in posterior carapace portion. Gastric region large, indistinctly demarcated with grooves. Cardiac region ovate in outline, strongly arched. Branchial regions broad. Intestinal region narrow without large spines. Sternum, pleon, pereiopods unknown.

**Occurrence.** The species is known only from its type locality at Wetzelsdorf (Austria).

**Remarks.** *Iphiculus eliasi* **sp. nov.** is closest to the extant *I. convexus* in terms of the morphology of the dorsal carapace. The latter species, however, has a relatively wider carapace closer to the anterior half and possesses more large round tubercles on the dorsal carapace, especially on the hepatic and gastric regions (Fig. 2A *versus* Fig. 2B). Another extant species, *I. spongiosus*, differs from *I. eliasi* **sp. nov.** by the presence of large anterolateral spines and the possession of dorsal carapace tubercles which are more projected outwards (Ng *et al.* 2008: fig. 79). Similarly, all fossil species of *Iphiculus* so far known, i.e. *I. granulatus* Morris & Collins, 1991, *I*. *miriensis* Morris & Collins, 1991, and *I. sexspinosus* Morris & Collins, 1991, have well-developed anterolateral spines (Morris & Collins 1991: figs 15, 14 and 17, respectively) which are absent in *I. eliasi* **sp. nov.**

*Iphiculus eliasi* **sp. nov.** also resembles some *Typilobus* species. *Typilobus kishimotoi* Karasawa, 1998, from the Miocene of Japan has similarly shaped carapace outline and possesses large rounded tubercles on the dorsal surface; but the tubercles are fewer than in *I. eliasi* **sp. nov.** (Fig. 3A vs. Karasawa 1998: fig. 2). Additionally, *T. kishimotoi* has two longitudinal ridges behind the median sulcus of the frontal region (Karasawa 1998: fig. 2.2b), a feature that is absent in the new species (Figs. 2A, 3A, 3C).

One specimen of *Iphiculus eliasi* **sp. nov.** (UMJGP 21.1339) exhibits partial cuticle degradation on the preserved portion of the carapace (Fig. 4A–C). It suggests rather complex internal structure of the tuberculation of leucosioids; tubercles are expressed differently in various cuticular layers. It was already noted that taphonomic aspects of the cuticle preservation in fossil crabs may have major impact on taxonomical evaluation of the characters such as carapace ornamentation (e.g. Feldmann & Portell 2007; Klompmaker *et al.* 2015). Therefore, only large rounded tubercles present also on the specimen without preserved cuticle (Fig. 3A–D) are considered of taxonomical importance herein.

Vía (1941) reported an “Iliinae, Ebaliinae?” from the Middle Miocene of Catalonia, Spain. His figure (Vía 1941: pl. 10, fig. 75) clearly represents a leucosioid crab with striking similarities to *Iphiculus eliasi* **sp. nov.** Müller (1993: figs 5M–N) refigured the specimen in greater detail (although still insufficient for closer comparison) and assigned it questionably to the leucosiid genus *Randallia* Stimpson, 1857 *sensu lato* (see Galil 2003). Re-examination of the specimen is needed to resolve its affinities.

## Notes on palaeobiogeography

Representatives of the family Iphiculidae are so far restricted to the Indo-West Pacific, with most taxa known from the Indo-Malaysian archipelago (Galil 2007; Galil & Ng 2007, 2009, 2010). Iphiculid crabs have occupied this area at least since the Middle Miocene (Morris & Collins 1991; Collins *et al.* 2003). At that time, however, their geographical distribution was probably wider, and included the circum-Mediterranean area as is evident by the occurrence of *Iphiculus eliasi* **sp. nov.** The recognition of *Iphiculus* in the Middle Miocene strata of Austria suggests that the genus originated in the Western Tethys and subsequently migrated into the Indo-West Pacific (“Go East!” concept of Harzhauser *et al.* 2007, 2008; or “Biodiversity Hopping Hotspots” concept of Renema *et al.* 2008) or contraction of originally broader geographic distribution due to tectonic (development of the *Gomphotherium* Landbridge; Rögl 1998, 1999; Harzhauser *et al.* 2007; see also Khodaverdi Hassan-vand *et al.* 2016) and/or climatic factors (post-Middle Miocene cooling events; Zachos *et al.* 2001, 2008). The biogeographic history of the Iphiculidae, and *Iphiculus* in particular, may be even more complex as collection bias cannot be ruled out. In fact, nearly all fossil occurrences of the Iphiculidae are restricted to Borneo, where major collecting efforts have been done due to various reasons (for details see Morris & Collins 1991; Collins *et al.* 2003).

## Figures and Tables

**Table 1:** Synopsis of the Iphiculidae. *I*. = *Iphiculus*; *P*. = *Pariphiculus*.

Taxon	Age	Occurrence	Reference(s)
*I. eliasi* **sp. nov**.	Middle Miocene	Austria	This paper
*I. sexspinosus* Morris & Collins, 1991	Late Miocene–Pliocene	Brunei, Sabah, Sarawak	Morris & Collins (1991), Collins et al. (2003)
*I. granulatus* Morris & Collins, 1991	Pliocene	Brunei	Morris & Collins (1991)
*I. miriensis* Morris & Collins, 1991	Pliocene	Brunei	Morris & Collins (1991)
*I. convexus* Ihle, 1918	Recent	Philippines to Vanuatu	Galil & Ng (2009, 2010)
*I. spongiosus* Adams & White, 1849	Recent	Red Sea to Vanuatu	Galil (2007), Galil & Ng (2010)
*P. decemtuberculatus* Collins et al., 2003	Middle–Late Miocene	Sabah	Collins et al. (2003)
*P. multituberculatus* Collins et al., 2003	Middle–Late Miocene	Sarawak	Collins et al. (2003)
*P. gselli* Beets, 1950	Middle Miocene	Java	Beets (1950)
*P. gselli beetsi* Morris & Collins, 1991	Middle Miocene–Pliocene	Brunei, Sarawak	Morris & Collins (1991), Collins et al. (2003)
*P. papillosus* Morris & Collins, 1991	Middle Miocene–Pliocene	Brunei, Sarawak	Morris & Collins (1991), Collins et al. (2003)
*P. inconditus* Karasawa, 1993	Pliocene	Japan	Karasawa (1993)
*P. verrucosus* Morris & Collins, 1991	Pliocene	Sarawak	Morris & Collins (1991)
*P. agariciferus* Ihle, 1918	Recent	Japan to Vanuatu	Galil & Ng (2007, 2010)
*P. coronatus* (Alcock & Anderson, 1894)	Recent	Japan to Solomon Is.	Galil (2007)
*P. mariannae* (Herklots, 1852)	Recent	Arabian Sea to Vanuatu	Galil (2007), Galil & Ng (2010)
